# A Longitudinal Study of the Development of Executive Function and Calibration Accuracy

**DOI:** 10.3390/children11030364

**Published:** 2024-03-19

**Authors:** Marios Goudas, Evdoxia Samara, Athanasios Kolovelonis

**Affiliations:** Department of Physical Education and Sport Science, University of Thessaly, 42100 Trikala, Greece; evdosamara@pe.uth.gr (E.S.); akolov@pe.uth.gr (A.K.)

**Keywords:** latent growth-curve modeling, metacognition, development, preadolescence, basketball shooting, performance estimation, design-fluency test, inhibition, shifting

## Abstract

This longitudinal study examined the development of executive function and calibration accuracy in preadolescents. This study’s sample consisted of 262 students (127 females) from grades 4 (*n* = 91), 5 (*n* = 89), and 6 (*n* = 82) who took measures of executive function and performance calibration in a sport task three times over 20 months. A latent growth-curve modeling analysis showed a significant relationship between the rates of change of executive function and calibration accuracy. The results also showed a dynamic interplay in the development of executive function and calibration accuracy. There were significant interindividual differences in the estimated population means both in executive function and calibration accuracy and in the rate of change of executive function, but not in the rate of change of calibration accuracy. The age of the participants had a positive effect only on the estimated population mean of executive function.

## 1. Introduction

Executive functions (EFs) and metacognitive processes are higher-order cognitive processes. The development of these processes and their relationships to effective functioning in everyday tasks, including academic ones, have been extensively examined [1,2]. However, despite the strong conceptual links between these two groups of processes [3], they have often been studied independently. As a result, there is little evidence of how their relationship progresses developmentally [4]. The present study aimed to add to the limited scholarship that simultaneously charts the development of metacognitive processes and EFs. In particular, the study focused on the developmental relations between EFs and the metacognitive process of performance estimations.

EFs are theorized as being higher-order cognitive processes that facilitate the cognitive adaptability and flexibility of goal-oriented behavior. They support engagement in well-planned future-oriented behavior [5], and they enable the coordination of cognitive processes required for formulating goals, developing plans to achieve them, and implementing these plans effectively [6]. EFs are assumed to be triggered in novel, challenging, and complex situations when concentration and attention are needed [7]. Based mainly on Miyake et al.’s [8] work, most scholars believe that there are three core EFs, namely, inhibition, working memory, and cognitive flexibility [7].

Following Diamond [7], inhibition allows students to block habitual thoughts and actions and control their attention, behavior, thoughts, and emotions, which enables them to focus on the task at hand and take the most appropriate actions. Working memory refers to the short-term storage and handling of information. It enables the interrelation and reorganization of pieces of information or the incorporation of new information into action plans. Cognitive flexibility, or shifting, allows students to change approaches when solving a problem while shifting attention between task demands and to adjust to new demands, rules, or priorities [7].

Health, quality of life, and success in school and life have been positively linked to EFs [1]. For example, studies by Alloway and Alloway [9], Roebers et al. [10], and Cantin et al. [11] have shown the positive effects of EFs on school success and academic achievement. Sport performance has also been linked to EFs [12,13,14].

The concept of metacognition (MC) was introduced by Flavell [15], who defined it as cognition of cognition that serves two basic functions, namely, the monitoring and control of cognition. Following Flavell, three facets of MC have been theorized: metacognitive knowledge, which refers to declarative knowledge regarding tasks, strategies, and goals; metacognitive experiences, which denote persons’ awareness of their feelings and perceptions when coming across a task; and metacognitive skills, which pertain to the deliberate use of strategies to control cognition [16].

Calibration refers to the degree of the fit between individuals’ judgments of performance and their actual performance [17]. Therefore, calibration is the difference between one’s estimated performance and actual performance on specific tasks [18]. This difference can be viewed as absolute accuracy (i.e., absolute fit between estimated and actual performance) or relative accuracy (i.e., discrimination of performance across items) [18]. Calibration involves awareness of internal processes [19], including monitoring what students know about a topic or skill and judging this knowledge with a criterion task. Thus, calibration is considered a metacognitive experience. The accuracy of these experiences is important for students’ performance and self-regulated learning as it influences their decisions about strategy use, effort exertion, and selection of future activities [19].

Well-calibrated students can improve their performance by focusing on aspects of tasks they have not mastered yet [20]. In contrast, miscalibrated students may set unrealistic or rather easy goals, which may prevent them from improving their performance [21]. Miscalibrated students may also be less responsive to external feedback (i.e., how to improve skills), and they may not take responsibility for their development due to inaccurate self-feedback regarding their learning.

Research in academic settings has shown that students usually overestimate their performance [22,23]. Similarly, in physical education, students overestimate their performance in sport tasks, such as basketball chest pass, basketball shooting, and soccer pass [24,25].

Scholars have noted a conceptual similarity between EFs and MC [3,26]. These concepts have been theorized as higher-order cognitive processes that allow individuals to function and adapt effectively to new and demanding tasks. Furthermore, they are seen as being initiated and controlled by the individual. Both involve subprocedures (e.g., shifting, updating, and inhibition for EFs, and monitoring and control for MC). Additionally, both concepts refer to the individuals’ ability to monitor and control their thinking and behavior. Given the theoretical overlap between these two notions, it is somewhat surprising that their development has been examined independently.

Regarding the development of EFs, a review by Best and Miller [27] found that inhibition showed a rapid and large improvement during preschool years and slower improvement later on. Working memory showed a linear and longer improvement between the preschool period and adolescence. Shifting developed later than the other two core EFs as it is based on them [28]. More recent longitudinal studies have generally confirmed the assertions of Best and Miller [25]. For example, Lee et al. [29] reported that working memory showed a steady increase from 6 to 15 years, while Brydges et al. [30] documented improvements in all three EFs from 8 to 10 years.

Concerning the development of calibration, scholars have indicated an improvement in calibration accuracy with age [31]. In one study [32], kindergartners showed a stronger overconfidence compared to second graders in computer-based learning tasks. In two other studies [33,34], early primary schoolers overestimated themselves in comparison to older children. In sport settings, a positive relationship between age and prediction accuracy was found among runners, with older runners showing better calibration accuracy than younger ones [35]. In physical education, sixth-grade students were more accurate compared to fifth-grade students [36]. Although the evidence of these cross-sectional studies suggests an improvement in calibration accuracy with age, there is a lack of related longitudinal research that charts the development of this metacognitive experience [37].

Roebers [3] summarized the results of studies on the development of EFs and MC. Regarding the former, she concluded that a significant improvement in inhibition and working memory appears between the third and fourth year of life, while continuous enhancement of these EFs as well as shifting takes place in middle childhood and adolescence. Similarly, concerning MC, remarkable improvements are observed around the fourth year of age, although several actions, including making performance predictions, are still difficult for upper-elementary school children [38].

Given the theoretical overlap between EFs and MC as well as developmental findings denoting a rapid improvement of both aspects during early life and a continuous enhancement during school years, it would be informative to study how their relationship progresses developmentally. This would help to better understand developmental mechanisms [39]. However, few studies have focused on the development of both EFs and MC. Howard et al. [4] assessed children’s EFs and metacognitive abilities at three time points: the beginning of children’s final preschool year, the end of the final preschool year, and the end of the first year of school. Their results show bidirectional associations between EFs and MC in a cross-lagged panel model. Spiess et al. [39] measured EFs and metacognitive control twice within eight months in eight-year-old children. Their results show an improvement in both EFs and MC; however, in contrast to Howard et al. [4], the longitudinal links between EFs and MC were not significant. This finding may be due to the stability of the constructs in the relatively short period of Spiess et al.’s study. Finally, in a study with first graders, Roebers et al. [10] reported that EFs were significantly related to MC both cross-sectionally and longitudinally.

The present study aimed to contribute to the scholarship that examines concurrently the development of EFs and MC. Investigating different aspects of cognitive development together is important because it can provide information regarding which aspect precedes others and help to understand developmental mechanisms [39]. This study asked the following research questions: (1) Is there a significant relationship between the rates of change of EFs and calibration, as well as between the latent scores of EFs and calibration? (2) Are there significant interindividual differences in the rates of change of EFs and calibration? (3) Does age affect the rates of change of EFs and calibration? This longitudinal study enriches the limited pool of relevant studies in three important ways. First, by employing latent-growth modeling, it assessed the rates of change in EFs and the metacognitive skill of calibration as well as the magnitude of the relationship between the two rates. Furthermore, it examined potential individual differences in these developmental trajectories. Second, the study focused on 10- to 12-year-old students because both EFs and MC are still developing in them, while the specific metacognitive skill of performance prediction is underdeveloped [38]. Third, as previous studies employed cognitive tasks to examine calibration [39], the present study used an authentic school-sport task to advance the literature.

## 2. Materials and Methods

### 2.1. Design

The present study adopted a longitudinal design involving two measures (EFs and calibration) taken at three time points. Assessments of EFs and calibration as part of a sport task were conducted at the start of the academic year (T1), at its end (T2), and one year later at the end of the next academic year (T3).

### 2.2. Participants

The participants were 262 grade 4, 5, and 6 students (127 females) from three elementary schools located in a medium-sized city in central Greece. Specifically, 91 students were from grade 4 (*M* age = 9.39, *SD* = 0.28, 45 females); 89 were from grade 5 (*M* age = 10.5, *SD* = 0.31, 43 females), and 82 were from grade 6 (*M* age = 11.41, *SD* = 0.26, 39 females). Of these, 249 pupils took part at T2 (grade 4: *n* = 87, grade 5: *n* = 82, grade 6: *n* = 80), while 233 were assessed at T3 (grade 4: *n* = 82, grade 5: *n* = 80, grade 6: *n* = 71). The students at T3 were attending the next grade compared to those at T1 and T2. The participants were free from medical problems. The sample size was determined to meet the sample-size requirements for latent-growth modeling [40].

### 2.3. Measures

#### 2.3.1. Executive Function

The design-fluency test [41,42], a component of the Delis–Kaplan Executive Function System, was administered to evaluate students’ EFs. The test includes three conditions. The first one evaluates fluency in generating visual patterns, the second one assesses inhibition, and the third one judges switching. The sum of a student’s scores in the three test conditions served as a measure of executive function.

#### 2.3.2. Sport Task

A basketball shooting test [43] was employed. This test has demonstrated satisfactory test–retest reliability (0.92). The participants had to shoot, without a time limit, 10 shots in front of the basket from a distance of 2.5 m. The height of the basket was 2.8 m. The score was the number of successful shots. This task has been used successfully in sport-related calibration research [24,25,44].

#### 2.3.3. Calibration

To gauge calibration accuracy, the students predicted their success rates out of 10 attempts in the basketball shooting task; then, their performance was measured. The participants answered the following question: “How many of your shots out of 10 will be successful from this position in the following test?”. The answers were the scores for the estimation of shooting performance. The calibration accuracy index (i.e., absolute values of the difference between estimated and actual performance) [18] was used. With this index, scores closer to zero indicate higher calibration accuracy.

### 2.4. Procedures

Institutional review board approval was obtained prior to the study’s commencement. Written parental consent was also obtained. Students were informed that their participation was optional and that their anonymity was guaranteed. The participants completed the design-fluency test in their classrooms. For each condition of the test, the experimenter provided instructions and demonstrated one trial on the classroom’s blackboard. The students performed a practice trial before proceeding to each condition of the test. Next, they provided estimations of their performance on the basketball test and took the test individually on the basketball courts of their schools.

### 2.5. Statistical Analysis

Latent-growth modeling analysis was performed with Mplus (version 8.1) [45]. Preliminary analyses involved descriptive statistics and correlations for the three time points, examination of the univariate kurtosis, and checking whether missing data were missing completely at random.

## 3. Results

### 3.1. Preliminary Analyses

The kurtosis values obtained for the accuracy and EF measures across the three measures were 2, 0.82, and 1.18, and 0.21, 0.30, and 0.21, respectively, which fall within acceptable ranges according to Byrne [40]. Although these univariate-kurtosis values were acceptable, following a suggestion by Byrne [40], the latent-growth modeling analyses were performed both with Mplus’s ML estimator, which is more sensitive to multivariate kurtosis, and with the MLM estimator, which provides more robust results in case of multivariate kurtosis. Given that the χ^2^ results of the two analyses did not differ substantially, based on Byrne [40], it is reasonable to infer that the dataset exhibited multivariate normality. Since the ML estimator uses all the cases, including those with missing data, in contrast to the MLM estimator, which uses only the cases with complete data, we present the results provided by the former.

Regarding the type of missing data, the students with complete data on the three measures did not differ significantly in terms of accuracy and EF at T1 from those who had missing data on these two variables either at T2 or T3 or at T2 and T3 (Wilks’s lambda = 0.994, F(2, 259) = 0.837, *p* > 0.01). Therefore, the data from all 262 students were used in the subsequent analyses.

### 3.2. Descriptives Statistics and Correlations

The descriptive statistics for the sample of participants are presented in Table 1; Pearson product–moment correlation coefficients between the variables of the study are shown in Table 2. Small improvements were found both in accuracy and EF across the three measures, while small correlations were observed between accuracy and EF.

### 3.3. Latent Growth Curve Modeling

**Figure 1:**
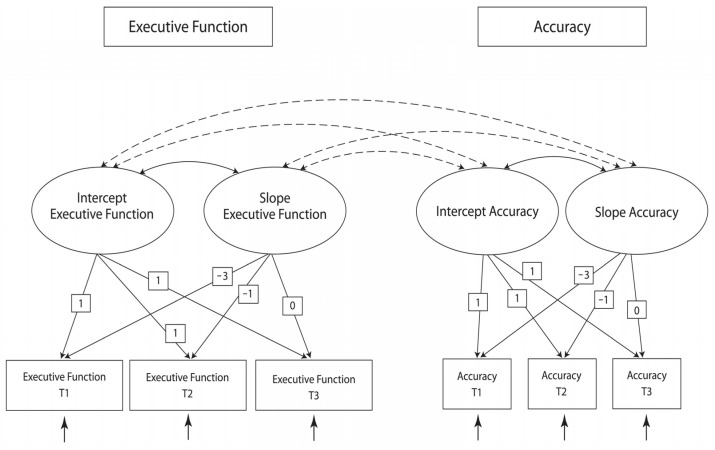
The hypothesized model (Model 1).

Latent-growth modeling permits the estimation of latent factors termed intercepts and slopes as well as their means and variances. Intercepts indicate an individual’s score at a specified time of measurement, while slopes represent the rate of change over the period of interest. The means of these latent factors represent estimations of the population scores at the time of interest (intercept) and the population’s trajectory of “true” change within the measurement period (slope), while their variances reflect population interindividual differences [40]. We defined a dual-domain linear-growth model (Figure 1), which involved six measured variables (total score on the design-fluency test and calibration measured at three time points), four latent factors (intercept and slope for each domain), and covariances between the latent factors. We centered the model at T3 to examine the estimated population mean and variance at that time.

**Figure 2:**
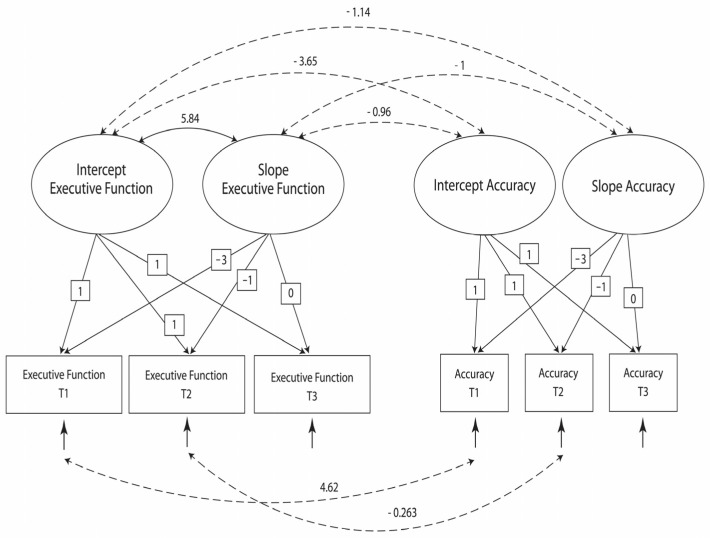
The revised model (Model 2).

This model exhibited nonacceptable fit indices (Table 3: Model 1). A revision of this model entailed the addition of two covariances between two pairs of error variances (based on the modification indices) and the elimination of the nonsignificant covariance between the accuracy intercept and the accuracy slope. The revised model presented acceptable fit indices (Table 3: Model 2, Figure 2).

Table 4 presents the covariances between the latent factors (Model 2). Both within-domain (intercept of accuracy with slope of accuracy) and between-domains (intercept of accuracy with slope of EF) significant covariances were observed.

Table 5 presents the means and variances of the latent factors. The estimated rate of improvement of calibration accuracy was not significant. In contrast, an improvement of 2.43 between T1 and T3 resulted in an estimated population mean of 27.67 at T3 for EF.

**Figure 3:**
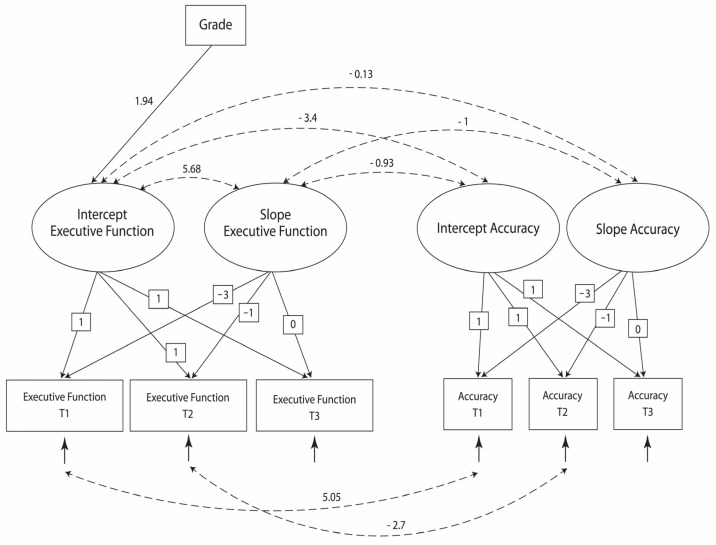
The model with grade as a predictor (Model 3).

Given the high variability of three out of four latent factors, which indicated high interindividual variability, we examined whether the inclusion of grade as a predictor variable could explain this variability (Model 3, Figure 3). The goodness-of-fit statistics (Table 3) for this model were adequate. The results suggested a significant effect of grade on the intercept of EF.

## 4. Discussion

This study examined the parallel development of EF and calibration accuracy through latent growth-curve modeling. This approach can provide insights regarding the change in constructs over time, including the relationship between the rates of change, as well as evidence concerning interindividual variability in the growth of the constructs.

With regard to the first research question, the findings show a significant relationship, which suggests that students who progressed in one of the constructs did so in the other one as well. A related noteworthy aspect of the results is the interrelationships between the rates of change and the intercepts of EF and calibration. The rate of change (slope) of EF correlated to the intercept of calibration. Given that calibration was centered at T3, this finding indicates that the participants with better calibration scores at T3 had a higher rate of change in EF. Conversely, the significant relationship of the intercept of EF with the rate of change (slope) of calibration shows that the students with higher EF scores at T3 had a higher rate of change in calibration. Taken together, these results suggest that the two concepts are linked and affect the development of each other. The findings of the present study confirm those of Howard et al. [4] but not those of Spiess et al. [39]. They also partially support the results of Roebers et al. [10], who tested and reported only a longitudinal direct influence of EF on MC. The different ages of the participants in the present study, as well as the difference in the metacognitive aspect examined and the tasks employed, may account for these conflicting findings.

Furthermore, concerning the relationship between EF and calibration accuracy, the results indicate a strong relation between the respective latent factors. This shows that at T3, the students with higher EF scores also had better calibration scores. Generally, previous correlational studies with children found low relationships between EFs and aspects of MC [46,47]. Similarly, in a study conducted with six-year-olds, Destan and Roebers [48] reported no associations between EFs and calibration accuracy. In contrast, when structural equation modeling was utilized, a substantial relationship between EF and aspects of MC in children was uncovered [10,39]. The results of the present study, which also employed this approach, point to a significant relation. As such, structural equation modeling procedures may show the “true” shared variance between metacognitive processes and EFs in the relevant studies [3].

The second research question pertained to the potential individual variability in the intercepts and slopes of the latent factors. Significant variances emerged for the latent factors of the intercepts of EF and calibration as well as the rate of change (slope) of EF. These variances suggest that there were significant interindividual differences at T3 both for EF and calibration as well as for the rate of change of EF. These differences may have been due to the combined effect of cognitive developmental factors and the students’ previous educational experiences [49].

Regarding the third research question, the addition of age as a possible factor that could account for the abovementioned interindividual differences showed that only EF at T3 was affected. Thus, at T3, the estimated population mean for EF was positively affected by the students’ age, with older students having also higher EF scores. The rate of change for both EF and calibration accuracy, as well as the intercept of calibration accuracy, were not affected by students’ age, possibly due to individual differences. Overall, the results concerning the second and third research questions imply that chronological age is generally an imprecise variable for studying development due to high interindividual variability.

The rate of change of calibration, as well as its variability, was nonsignificant. Also, this rate was not affected by the students’ age. These results indicate that during the period under observation, the participants’ mean calibration did not change significantly, with no signs of interindividual differences. Although a recent meta-analysis [50] found that children’s self-overestimation gradually decreased with age from early to late childhood, this finding was based on cross-sectional studies. In contrast, the present study’s longitudinal results show that for the age range considered here, overestimation remained relatively stable.

A limitation of this study regards the instrument used for the measurement of EF. The design-fluency test does not assess working memory; it is only suitable for inhibition and switching. Therefore, future studies could use EF tests that measure working memory.

Overall, the findings of the present study add to the limited literature that has jointly examined the development of EF and aspects of MC [4,39]. This study found a dynamic interplay in the development of EF and calibration in students aged 10–12 years. Given that previous scholarly works [4,39] used different measurement tools for both EF and metacognitive aspects, no definite conclusions can be drawn concerning the developmental interaction of EF and metacognitive processes.

## Figures and Tables

**Table 1:** Descriptive statistics of the variables.

	T1		T2		T3	
	*Μ*	*SD*	*M*	*SD*	*M*	*SD*
Total sample						
Calibration accuracy	2.21	1.76	2.15	1.81	2.06	1.68
Executive function	20.30	7.23	26.10	8.23	27.02	8.61
Grade 4						
Calibration accuracy	2.86	2.16	2.31	1.97	2.21	1.95
Executive function	19.19	6.91	25.47	7.56	24.21	7.33
Grade 5						
Calibration accuracy	1.89	1.55	2.33	1.81	2.09	1.53
Executive function	20.87	7.27	25.38	8.06	28.63	8.95
Grade 6						
Calibration accuracy	1.83	1.22	1.80	1.58	1.85	1.48
Executive function	20.93	7.47	27.53	9.00	28.46	8.88

**Table 2:** Pearson’s *r* correlations between executive function and calibration.

T1	T2	T3
Total sample		
−0.14* 95% CI [−0.26, −0.019]	−0.35** 95% CI [−0.45, −0.24]	−0.25** 95% CI [−0.37, −0.13]
Grade 4		
−0.29** 95% CI [−0.47, −0.092]	−0.45** 95% CI [−0.6, −0.27]	−0.19 95% CI [−0.39, 0.028]
Grade 5		
−0.00 95% CI [−0.21, 0.21]	−0.39** 95% CI [−0.56, −0.19]	−0.41** 95% CI [−0.58, −0.21]
Grade 6		
0.01 95% CI [−0.12, 0.31]	−0.18 95% CI [−0.04, 0.38]	−0.14 95% CI [−0.096, 0.36]

* *p* < 0.05, ** *p* < 0.001.

**Table 3:** Goodness-of-fit indices.

	χ^2^/df	CFI	TLI	RMSEA	SRMR
Model 1	3.59	0.91	0.80	0.1	0.05
Model 2	1.91	0.97	0.93	0.06	0.04
Model 3	1.59	0.98	0.94	0.05	0.04

CFI: Comparative Fit Index, TLI: Tucker-Lewis Fit Index, RMSEA: Root Mean Square Error of Approximation, SRMR: Standardized Root Mean Square Residual.

**Table 4:** Covariances between the latent factors.

	1	2	3
1. Intercept of accuracy			
2. Intercept of executive function	−3.65 **		
3. Slope of accuracy		−1.14 *	
4. Slope of executive function	−0.96 **	5.85 *	−1 **

* *p* < 0.05, ** *p* < 0.001.

**Table 5:** Estimated means and variances of the latent factors.

	*M*		Variance	
	Estimate	SE	Estimate	SE
Intercept of accuracy	2.1 **	0.1	0.44 *	0.16
Slope of accuracy	−0.06	0.5	0.12	0.07
Intercept of executive function	27.67 **	0.52	39.24 **	6.67
Slope of executive function	2.43 **	0.19	4.94 *	1.80

* *p* < 0.05, ** *p* < 0.001.

## Data Availability

The data presented in this study are available on request from the corresponding author. The data are not publicly available due to privacy restrictions.
